# Moving-source elastic wave reconstruction for high-resolution optical coherence elastography

**DOI:** 10.1117/1.JBO.21.11.116006

**Published:** 2016-11-08

**Authors:** Bao-Yu Hsieh, Shaozhen Song, Thu-Mai Nguyen, Soon Joon Yoon, Tueng T. Shen, Ruikang K. Wang, Matthew O’Donnell

**Affiliations:** a University of Washington, Department of Bioengineering, 3720 15th Avenue NE, P. O. Box 355013, Seattle, Washington 98105, United States; b China Medical University, Department of Biomedical Imaging and Radiological Science, 91 Hsueh-Shih Road, Taichung 40402, Taiwan; c University of Washington, Department of Ophthalmology, 325 9th Avenue, Seattle, Washington 98104, United States

**Keywords:** presbyopia, crystalline lens, elasticity imaging, acoustic radiation force, moving-source reconstruction, optical coherence elasticity imaging

## Abstract

Optical coherence tomography (OCT)-based elasticity imaging can map soft tissue elasticity based on speckle-tracking of elastic wave propagation using highly sensitive phase measurements of OCT signals. Using a fixed elastic wave source and moving detection, current imaging sequences have difficulty in reconstructing tissue elasticity within speckle-free regions, for example, within the crystalline lens of the eye. We present a moving acoustic radiation force imaging sequence to reconstruct elastic properties within a speckle-free region by tracking elastic wave propagation from multiple laterally moving sources across the field of view. We demonstrate the proposed strategy using heterogeneous and partial speckle-free tissue-mimicking phantoms. Harder inclusions within the speckle-free region can be detected, and the contrast-to-noise ratio slightly enhanced compared to current OCE imaging sequences. The results suggest that a moving source approach may be appropriate for OCE studies within the large speckle-free regions of the crystalline lens.

Presbyopia is a long-sightedness disease resulting from the stiffening of the human crystalline lens with age, causing a gradual decrease in accommodation in nearly the entire population starting about age 40.^1^ Currently, there is no noninvasive tool that can map the heterogeneous elastic properties of the lens interior. In addition, as new minimally invasive and noninvasive procedures are considered for lens modification, a noninvasive tool is needed to help guide these procedures based on a personalized biomechanical model.^2^ Here, we investigate if such a tool can potentially be developed based on optical coherence elastography (OCE), a technique for mapping the elastic modulus in a medium at high spatial resolution using optical coherence tomography (OCT).

Shear wave elasticity imaging (SWEI) can provide quantitative and repeatable measurement of tissue stiffness in a clinical environment.^3^^–^^6^ It requires two steps: (1) remotely generating mechanical movement to create a shear wave source and (2) tracking shear wave propagation over space and time with an appropriate imaging system, such as MRI, ultrasound, or phase-sensitive optical coherence tomography (PhS-OCT). The resultant displacement map with time can be reconstructed with PhS speckle tracking and the local propagation velocity can be calculated based on a time-of-flight technique.

A map of the elastic modulus can be directly reconstructed from estimated group velocity at each point within an image according to the expression:^7^

$$V_{s}=\sqrt{\frac{\mu }{\rho }},$$ (1)

where μ is the local shear modulus, $V_{s}$ is the local shear wave propagation speed, and ρ is the local mass density. In nearly incompressible soft tissue, the Young’s elastic modulus is simply three times the shear modulus. Thus, tissue elasticity can be directly computed in a region from the reconstructed shear wave velocity in that region.

Ultrasound SWEI has been applied to a wide range of clinical applications, such as the liver,^8^ muscle,^9^ heart,^10^ and blood vessels.^11^ PhS speckle tracking can also be performed with PhS-OCT for OCT-based elastography, or OCE, with higher sensitivity for motion detection because of the greatly decreased wavelength of the probing beam and its high spatial resolution (typically ∼10  μm). Previous studies have shown that this technique can be used to assess the elasticity of skin^12^ and intraocular tissues^13^ with a mechanical vibrator as the shear source.

To assess the elasticity of the cornea, we recently presented a dynamic OCE system combining an acoustic radiation force (ARF)-based shear source and PhS-OCT to track displacement. The imaging technique produced high-resolution maps of elasticity in both tissue mimicking phantoms and cornea.^14^^–^^16^ In addition, a fully noncontact approach has been demonstrated in which a transient mechanical wave is launched by absorption of a single UV laser pulse, i.e., photoacoustic excitation, and tracked with ultrafast OCT for all-optical shear wave imaging on *ex-vivo* porcine cornea.^17^

In general, both ultrasound and OCT-based SWEI using a limited number of shear sources with moving-detector tracking provides quantitative and repeatable measurement of elastic properties within highly scattering tissue. The previous literature^18^ did provide an OCE-based method combining ARF and PhS-OCT to assess aged-related changes in the crystalline lens *in situ*. Either maximum displacement or model-based temporal analysis identifies age-related changes. However, the displacement can only be tracked on the lens surface, which has significant speckle, and the measurement appears to be a point detection even though it reflects the average elastic properties of the whole lens. The elasticity of the lens, including the cortex and nucleus, is fundamentally heterogeneous. For this relatively transparent tissue producing very low amplitude OCT signals (i.e., low echogenicity and even truly speckle-free regions), however, it is difficult for OCT to measure tissue displacement because the signal-to-noise ratio (SNR) is too low to reliably extract useful phase information. For this reason, it is almost impossible for OCT to track displacements within the interior region of the crystalline lens in the eye. Therefore, a different technique is needed to map the heterogeneous elastic modulus within the interior of the lens. Here, we present a potential solution using moving-source OCE to assess the elastic properties within weakly scattering or truly speckle-free regions.

Shear wave generation, propagation, and detection can be considered a linear process. Thus, the roles of source and detector can be interchanged without affecting the recorded signal. By exploiting this reciprocity principle, McAleavey et al.^19^ demonstrated that the same elastic properties could be obtained either by sweeping the source of shear waves (i.e., moving ARF or mARF) and keeping the detector fixed or by keeping the source fixed and sweeping the detector beam, as is done in SWEI, in general. For moving-detector OCE, a single source is utilized to create shear waves and moving detection is performed with M-mode acquisition for each position of a B-mode scan. However, there is no phase (speckle) information extracted from speckle-free regions, which means neither displacement nor shear wave propagation can be tracked over this area.

On the other hand, in moving-source reconstruction, multiple shear sources are used to generate shear waves, while M-mode data at a specific lateral position within a believable speckle region are acquired to track shear waves for each push and concatenated sequentially based on the distance between the shear source and the detection beam. Even if there is no speckle along the propagation path except at the detection position, each shear wave still can be tracked based on the time-of-flight from each shear source position to the single detection position. In addition, because of the details of the detection and generation mechanisms, the contrast-to-noise ratio (CNR) in shear wave images using a moving source compared to a conventional moving detector approach can also be slightly enhanced.^20^ Here, we explore an alternative quantitative strategy, moving-source OCE based on a moving ARF elastic wave source with PhS-OCT, which potentially can map the heterogeneous elastic modulus within the interior of the lens, as discussed below.

A 5-MHz linear ultrasound array (ATL L7-4, Philips Healthcare, Andover, Massachusetts) interfaced with a programmable US system (V1, Verasonics, Redmond, Washington) was used as a mARF source for electronically controlled elastic wave generation. For conventional moving-detector SWEI, a single push beam was applied to launch an elastic wave [Fig. 1(a)], while for a moving-source sequence, excitation beams were sequentially stepped laterally in 0.3-mm steps [Fig. 1(b)] for 64 beams covering a 19.2-mm wide field-of-view.

**Fig. 1:**
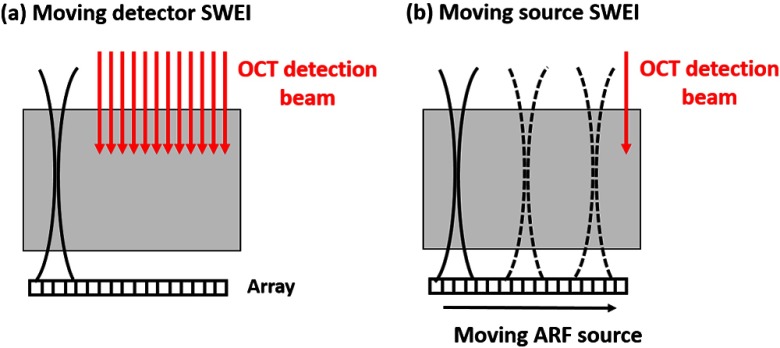
Schematic for (a) moving detector SWEI and (b) moving source SWEI.

The elastic wave remotely launched by ARF was then tracked with a PhS-OCT system.^14^ The light source of the PhS-OCT system is a super-luminescent diode with a central wavelength of 1310- and a 46-nm spectral bandwidth (half-maximum full-width of Gaussian profile). The focal length of the OCT probe is 110 mm, and the effective FWHM focal spot size is 52  μm. In the experimental setup used to demonstrate the principles of moving-source OCE, the ultrasound array delivered the push beam from the bottom of the phantom and the OCT tracking beam was positioned from the top, as shown in Fig. 1. The push duration, focal length, and acoustic f-number were 12.8  μs, 25 mm, and 1, respectively.

After the ultrasound push, the displacement associated with elastic wave propagation was detected by the PhS-OCT imaging system operating in an M-B scanning mode, which means that a high-speed 125-kHz A-line rate acquired M-mode data along one beam line over a dwell time of 4.8 ms, and then that beam line was sequentially scanned laterally by a 1-D scanning galvo mirror to acquire M-mode data for each position over the B-scan sweep. The scan parameters were the same as the US system such that 64 OCT beams were used to cover a 19.2-mm field-of-view, which limited the lateral pixel size to 0.3 mm. The theoretical axial resolution of the OCT system calculated from the central wavelength and spectrum width is 16.5  μm in air, and the actual resolution is measured as 21  μm from the point-spread-function captured with a mirror. A detailed description of the system can be found in a previous publication.^15^ All OCT imaging data were processed using the algorithm described in Ref. 16 to remove surface ripple artifacts commonly present in OCT shear wave imaging.

Two tissue-mimicking phantoms were designed for these studies. First, we made a heterogeneous gelatin phantom with a stiffer inclusion embedded inside to compare the imaging performance of moving-source reconstruction with the conventional moving-detector approach. The bulk phantom was made with a mixture of 6% w/w gelatin and 0.02% w/w titanium dioxide (${\mathrm{TiO}}_{2}$) acting as optical scatterers, while the stiffer inclusion was made with a mixture of 10% w/w gelatin and 0.02% ${\mathrm{TiO}}_{2}$. The phantom design is shown in Fig 2(a). A second design was used to produce a partially speckle-free phantom, as shown in Fig. 3(a). The left side was made with 6% w/w pure gelatin without any scatterers but with a stiff inclusion containing 10% w/w gelatin embedded inside. A drop of dye was added to easily visualize the inclusion for proper alignment with the imaging system. The right side of the phantom was made with 6% w/w gelatin and 0.02% w/w ${\mathrm{TiO}}_{2}$.

**Fig. 2:**
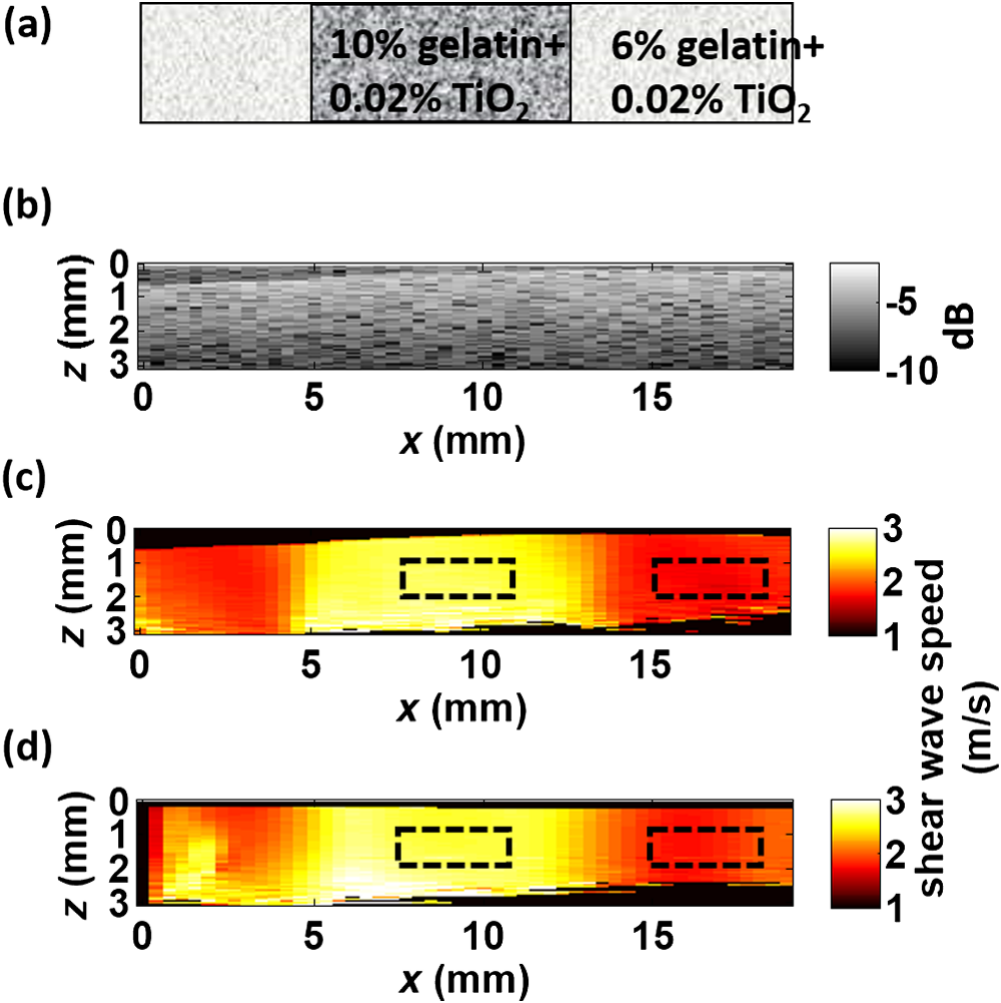
OCT-SWEI: (a) designed sketch, (b) B-mode, (c) elastic wave speed map of moving-detector SWEI, and (d) elastic wave speed map of moving-source SWEI of heterogeneous phantom. [Dashed line box in Figs. 2(c) and 2(d): a region defined to compute CNR].

**Fig. 3:**
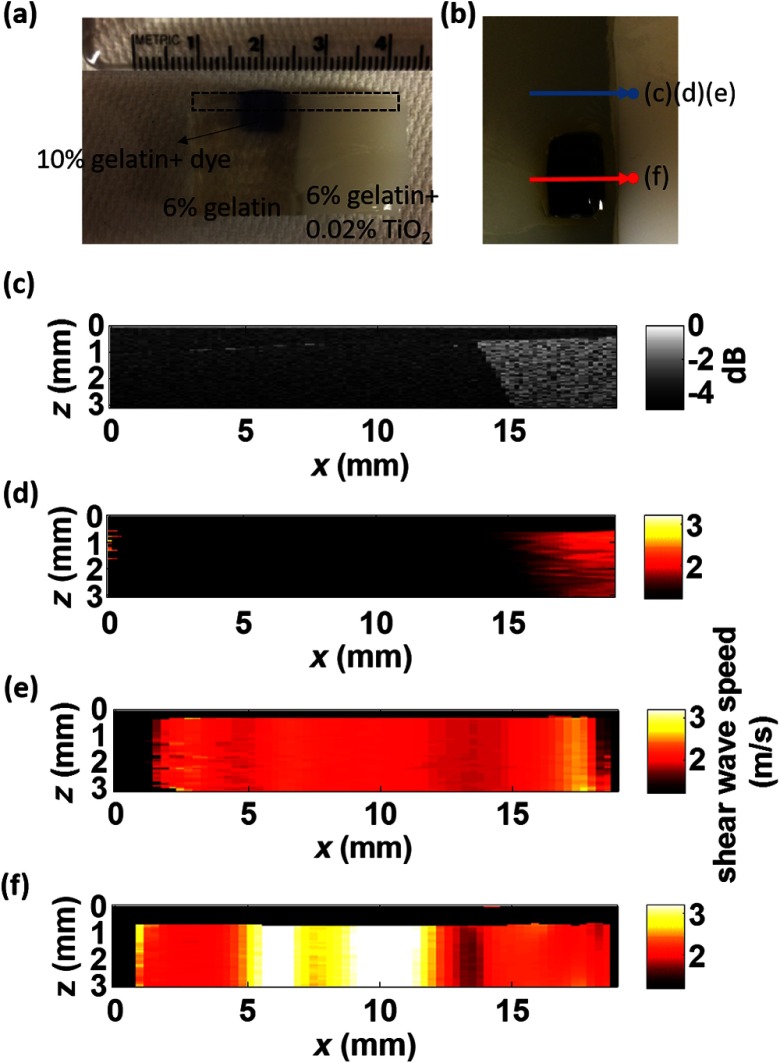
OCT-SWEI: (a) cross-section view, (b) top view of designed imaging phantom for OCT imaging, (c) OCT B-mode image, elastic wave speed map with (d) moving detector SWEI, (e) moving source SWEI in homogeneous, and (f) in heterogeneous part of phantom. [Dashed line box in Fig. 3(a) indicates the imaging field of view for Figs. 3(c)–3(f); scan line for (c), (d), (e): blue arrow and scan line for (f): red arrow].

This phantom was analyzed using both forms of SWEI to compare the quality of reconstructed elastic wave speed maps. The total length and width of both bulk phantoms are around 50 mm, but the region analyzed depended on the field-of-view of the OCE system, which is only 19.2 mm in length. Since the total height of the phantom is 30 mm and the focal length of the transducer attached to the bottom surface is 25 mm, the ultrasound excitation focus extends over the entire depth of the phantom and is strongest over the middle 10 to 15 mm of the sample.

To compare moving-source and moving-detector approaches, full M-B mode acquisition was applied for each push beam at different lateral positions of the OCT imaging system to obtain a complete dataset of all possible source and detector positions. For moving-source reconstruction, M-mode data at a specific lateral position within a speckle region were extracted from the dataset for each push and concatenated based on the distance between the elastic wave source and the detection beam. For moving-detector reconstruction, multiple detection beams were used to track axial displacements associated with elastic wave propagation from a single source. Only one measurement is acquired per experiment (i.e., no signal averaging).

Local displacements were calculated using the phase-zero-crossing of the cross-correlation along depth.^21^ A directional filter was then applied to remove reflection artifacts. The elastic wave speed map, corresponding to the shear modulus, is then reconstructed with a time-of-flight algorithm.^22^ To compare image quality for potential lesion detection, the CNR for both reconstructed images was calculated using

$$\mathrm{CNR}=\frac{\left|V_{\text{in}}-V_{\text{out}}\right|}{\sqrt{\sigma_{\text{in}}^{2}+\sigma_{\text{out}}^{2}}},$$ (2)

where $V_{\text{in}}$ and $\sigma_{\text{in}}$ are the mean wave speed and standard deviation in the inclusion, and $V_{\text{out}}$ and $\sigma_{\text{out}}$ are the mean wave speed and standard deviation outside of the inclusion.

The OCT structural image of the first phantom is shown in Fig. 2(b). Figures 2(c) and 2(d) show the reconstructed wave speed images using both imaging sequences, moving-detector and moving-source OCE, respectively. The embedded inclusion is not obvious in the B-mode image, but the elastic wave speed map from moving-source OCE [Fig. 2(d)] highlights the stiff inclusion similar to the one from moving-detector OCE [Fig. 2(c)]. Using the areas noted by the boxes in Fig. 2, the CNR for the inclusion was computed for moving-detector reconstruction and compared to that computed for moving-source reconstruction. The moving source reconstruction had a slightly higher CNR by 1.1 dB.

The primary reason for the enhanced CNR of the swept source sequence is the lower noise level resulting from the common OCT speckle region used for tracking; that is, the OCT speckle pattern is common for all moving-source measurements but is different for all moving-detector measurements since speckle patterns are almost completely uncorrelated laterally in an OCT image. This effect has been identified in ultrasound speckle tracking as well.^20^ Variations in the reconstructed elastic wave speed are generally lower and smoother in the moving-source image [Fig. 2(d)] than in the moving-detector image [Fig. 2(c)], except for the artifact on the left side of the image [Fig. 2(d)]. This arises when the push and detector positions are very close to each other and the acoustic wave induces a significant artifact in the OCT image.

Both imaging sequences were also tested on the partially speckle-free phantom, as shown in Fig. 3(a) in the cross section and from the top in Fig. 3(b). Figure 3(c) shows the OCT B-mode image of this phantom close to the boundary. Clearly, there is no speckle signal on the left, where phase extraction for displacement estimation is not reliable for tracking elastic wave propagation, as shown in Fig. 3(d). However, Figs. 3(e) and 3(f) show the reconstructed elastic wave speed maps of homogeneous [blue arrow in Fig. 3(b)] and heterogeneous [red arrow in Fig. 3(b)] regions of the partially speckle-free phantom with moving-source reconstruction. The slope boundary in Figs. 3(e) and 3(f) does not appear in the OCE image because the elastic modulus on both sides of that boundary is constant (i.e., there is an edge in optical scattering but not one in elastic modulus). Not only can the elasticity within the speckle-free region be reconstructed but also the heterogeneity of the phantom can be identified with high contrast.

Moving-source OCE is a variant of conventional moving-detector OCE that can estimate elastic properties in speckle-free regions with potentially high CNR.^20^ However, there are still trade-offs between acoustic exposure and image acquisition time for moving-source OCE because multiple stimulations are needed. Also, the elastic waveforms created by each stimulation at different spatial locations with varied elastic properties may differ because of variations in the ARF patterns. This may cause some artifacts, like the enhanced boundary in Fig. 3(f). In addition, if only a single detector (moving-source) or a single source (moving-detector) is used for data acquisition, then reconstructions are produced only for wave propagation in a single direction. Directional filters cannot completely identify forward and backward propagating waves, so for single direction reconstruction, artifacts will remain because of incomplete separation of these two components. Averaging reconstructions acquired over multiple source and detector positions will minimize these artifacts. Consequently, future work will focus on developing a hybrid sequence between moving-source and moving-detector approaches to optimize image CNR and minimize artifacts. Such an approach will most probably contain a limited number of source and detector positions to reconstruct the elastic modulus in speckle-free regions, such as the body of the crystalline lens.

By measuring the dispersion of shear wave speed in a viscoelastic medium, the shear modulus can be quantified from the real part of the complex modulus. Dispersion curves can be fit to a proper tissue model to quantify the complex modulus of the medium. Kelvin–Voigt is a widely used tissue model and the shear wave dispersion is given by the expression: $V_{s}={\left(2(\mu_{1}^{2}+\omega^{2}\mu_{2}^{2})/\{\rho [\mu_{1}+{\left(\mu_{1}^{2}+\omega^{2}\mu_{2}^{2}\right)}^{0.5}]\}\right)}^{0.5}$, where the wave speed is generally higher than that predicted from the simple expression, $V_{s}=\sqrt{\mu /\rho }$, when viscosity is taken into account.^23^ From our reconstructed images shown in Fig. 3(f), the mean velocity of elastic wave propagation in the homogeneous phantom made with 10% gelatin is 2.79  m/s. Using the simple relation between wave speed and modulus, $V_{s}=\sqrt{\mu /\rho }$, the Young’s modulus of the 10% gelatin phantom is 23.34 kPa. Compared to the value from previous work,^24^ our estimation is slightly larger than that (20.6 kPa) measured by an air-pulse-OCT system. Viscosity is not taken into account and we operate at higher mechanical wave frequencies than the air-puff system, so a slightly higher modulus estimate is reasonable. Also, to demonstrate that moving-source reconstruction can characterize the elastic properties of the speckle-free region, the phantom is simply designed as a thick bulk phantom without any curvature and with an air-gelatin boundary on both sides. For real clinical applications, the curvature and thickness of the crystalline lens must be taken into account.^25^^–^^30^

In this study, to simplify alignment between the ultrasound excitation beam and OCT detection beam, they were positioned on opposite sides of the phantom so that the large ultrasound array transducer did not block the optical beam used for OCT scanning. For real clinical use, the system must be redesigned so that both optical and ultrasound beams propagate into the lens through the same surface (i.e., the cornea).

Figure 4(a) shows an OCT image obtained in our lab of a monkey crystalline lens. The central part is formed by lens fibers arranged in concentric layers and does not produce significant speckle in the OCT image. The lens capsule and epithelium surrounding the lens, however, contribute to a speckle signal with sufficient SNR to extract phase information for displacement measurement. In future studies, we will develop a comprehensive sequence integrating moving-source and moving-detector approaches to acquire shear wave information from the surrounding speckle, as shown in Fig. 4(b), to investigate biomechanical properties within the crystalline lens.

**Fig. 4:**
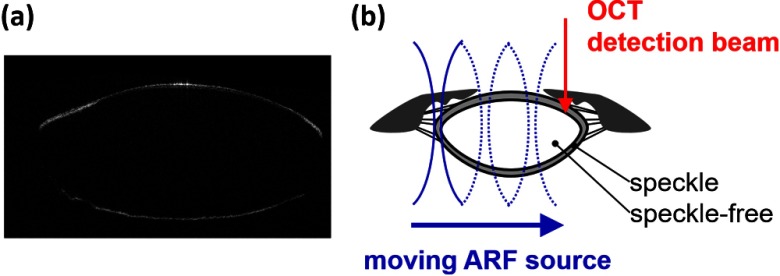
OCT-SWEI of the lens: (a) OCT image of a monkey’s crystalline lens and (b) moving beam approach for the crystalline lens.

In summary, we have demonstrated that the elastic properties of a speckle-free region can be reconstructed in simple phantoms by moving-source OCE. In addition, we have shown that this strategy provided slightly higher CNR (1.1-dB enhancement) compared with a conventional moving-detector reconstruction. These preliminary results suggest that a moving-source OCE sequence may be useful to quantitatively assess the elasticity in speckle-free regions with slightly higher CNR than a conventional moving-detector approach. A moving-source sequence potentially can map the heterogeneous elastic modulus within the interior of the lens where there is no speckle in OCT images. This approach will be tested in additional studies on the crystalline lens *in situ* and *in vivo*.
